# Acute Myeloid Leukemia Cells Functionally Compromise Hematopoietic Stem/Progenitor Cells Inhibiting Normal Hematopoiesis Through the Release of Extracellular Vesicles

**DOI:** 10.3389/fonc.2022.824562

**Published:** 2022-03-16

**Authors:** Stefania Trino, Ilaria Laurenzana, Daniela Lamorte, Giovanni Calice, Angelo De Stradis, Michele Santodirocco, Alessandro Sgambato, Antonella Caivano, Luciana De Luca

**Affiliations:** ^1^ Laboratory of Preclinical and Translational Research, Centro di Riferimento Oncologico della Basilicata (IRCCS-CROB), Rionero in Vulture, Italy; ^2^ Institute for Sustainable Plant Protection, National Research Council (CNR), Bari, Italy; ^3^ Trasfusional Medicine Department, Puglia Cord Blood Bank (CBB), Casa Sollievo Della Sofferenza Hospital, San Giovanni Rotondo, Italy; ^4^ Scientific Direction, Centro di Riferimento Oncologico della Basilicata (IRCCS-CROB), Rionero in Vulture, Italy; ^5^ Unit of Clinical Pathology, Centro di Riferimento Oncologico della Basilicata (IRCCS-CROB), Rionero in Vulture, Italy

**Keywords:** acute myeloid leukemia, hematopoiesis, extracellular vesicles, hematopoietic stem progenitor cells, microRNAs, differentiation, clonogenicity, inflammatory cytokines

## Abstract

Acute myeloid leukemia (AML) is an aggressive and heterogeneous clonal disorder of hematopoietic stem/progenitor cells (HSPCs). It is not well known how leukemia cells alter hematopoiesis promoting tumor growth and leukemic niche formation. In this study, we investigated how AML deregulates the hematopoietic process of HSPCs through the release of extracellular vesicles (EVs). First, we found that AML cells released a heterogeneous population of EVs containing microRNAs involved in AML pathogenesis. Notably, AML-EVs were able to influence the fate of HSPCs modifying their transcriptome. In fact, gene expression profile of AML-EV-treated HSPCs identified 923 down- and 630 up-regulated genes involved in hematopoiesis/differentiation, inflammatory cytokine production and cell movement. Indeed, most of the down-regulated genes are targeted by AML-EV-derived miRNAs. Furthermore, we demonstrated that AML-EVs were able to affect HSPC phenotype, modifying several biological functions, such as inhibiting cell differentiation and clonogenicity, activating inflammatory cytokine production and compromising cell movement. Indeed, a redistribution of HSPC populations was observed in AML-EV treated cells with a significant increase in the frequency of common myeloid progenitors and a reduction in granulocyte-macrophage progenitors and megakaryocyte-erythroid progenitors. This effect was accompanied by a reduction in HSPC colony formation. AML-EV treatment of HSPCs increased the levels of CCL3, IL-1B and CSF2 cytokines, involved in the inflammatory process and in cell movement, and decreased CXCR4 expression associated with a reduction of SDF-1 mediated-migration. In conclusion, this study demonstrates the existence of a powerful communication between AML cells and HSPCs, mediated by EVs, which suppresses normal hematopoiesis and potentially contributes to create a leukemic niche favorable to neoplastic development.

## Introduction

Acute myeloid leukemia (AML) is a neoplastic disorder characterized by an uncontrolled increase of myeloid precursors and their accumulation in both peripheral blood (PB) and bone marrow (BM) (1). AML is the result of hematopoietic stem or progenitor cell transformation through the acquisition of different genetic mutations and chromosomal rearrangements that induce their differentiation block and an increased proliferation (2, 3).

Despite the current available therapeutic approaches, such as chemotherapy and allogeneic hematopoietic stem cell transplantation, AML patient outcome remains unsatisfactory with more than half of patients dying from disease (1). Therefore, it is important to study the key mechanisms driving disease pathogenesis to design new and more effective pharmacological and therapeutic strategies.

Rapid clonal expansion of malignant cells in AML, occurring within the BM microenvironment, causes a replacement of heterogeneous hematopoietic and stromal cells, and impairs normal hematopoiesis and immune cells development (4). The abnormally expanded malignant blasts, not only benefit from BM niche, but also perturb it in order to induce a favorable microenvironment for leukemic progression (4–6).

Although literature data suggest that BM transformation by AML cells reduces the niche’s ability to retain hematopoietic stem/progenitor cells (HSPCs) and normal hematopoietic activity, a fine mechanism responsible for the modification of normal HSPC functions by leukemic cells remains elusive (7, 8).

Extracellular vesicles (EVs), lipid bilayer particles heterogeneous in term of biogenesis, size and content, are released from both normal and neoplastic cells (9). These particles are classified as small EVs, ranging from 30 to 200 nm, and medium/large EVs, ranging from 200 nm to 10 µm (10). EVs carry part of DNA, RNA, proteins, lipids and metabolites of the origin cells and are important players in both short- and long-range intercellular communication. In fact, by delivering their bioactive cargo, EVs can influence recipient cell function and behavior (5). In particular, neoplastic EVs are capable to support cancer growth and to disrupt healthy tissue homeostasis (11).

In hematological malignancies, neoplastic EVs promote tumor progression supporting auto-sustainability, through an autocrine loop and increasing aggressiveness (5).

Moreover, it has been demonstrated that EVs released by AML cells could participate in the regulation of BM function (12). In fact, in leukemic context, AML-EVs can reprogram mesenchymal stem cells (MSCs) and stromal cells, and down-regulate niche retention factor stromal cell-derived factor 1 (SDF1), resulting in mobilization of HSPCs from BM. Both AML and myelodysplastic syndrome cells were shown to reduce the hematopoiesis-supportive capacity of MSCs by delivering miR-7977 *via* EVs (13, 14).

In addition, it has been reported that exosomes, nanometer-size small EVs, can transport cargo from AML cells to BM stem cells contributing to the formation of a leukemia-supportive BM microenvironment (15, 16).

All these data suggest that both exosomal microRNA (miRNA) transfer and translational suppression of their target mRNAs might represent one of the possible explanations for the leukemia-associated loss of HSPC function and, consequently, for the inhibitory effect on hematopoiesis (7, 17).

Assuming that leukemic EVs may contribute to hematopoiesis impairment, in this study, we used umbilical cord blood (UCB) hematopoietic stem cells as a model to elucidate the effect of AML derived-EVs on HSPCs. In particular, we analyzed phenotype and miRNA content of AML EVs, gene profile of HSPCs and their biological functions, like differentiation, clonogenicity and cell movement, after *in vitro* AML-EV exposure.

## Matherials and Methods

### Cell Culture

The human AML cell lines, KG-1 and ME-1, were acquired from American Type Culture Collection (Rockville, MD, USA) and Deutsche Sammlung von Mikroorganismen und Zellkulturen (Braunschweig, Germany), respectively. Cells were cultured in RPMI-1640 medium (Gibco, Life technologies, Carlsbad, CA, USA) supplemented with 10% fetal bovine serum (FBS, Gibco), 1% of penicillin-streptomycin (Gibco) and 2 mM of L-glutamine (Gibco) at 37°C and 5% CO_2_. KG-1 AML cells were positive for CD4, CD11b, CD13, CD15, CD33, CD34, CD38 and CD90^+/-^, and negative for CD3, CD14, CD19 and CD117; ME-1 AML cells were positive for CD4, CD13, CD14^+/-^, CD33 and CD34, and negative for CD3, CD15 and CD19.

### EV Isolation From Cell Culture Medium

To obtain KG-1 and ME-1 derived EVs, 200x10^6^ cells were cultured at a density of 1.2x10^6^ cells/mL of RPMI-1640 medium without FBS for 48 h. Supernatant was collected and centrifuged firstly at 300 × g for 10 min and EVs were isolated as previously reported (18). After washing with 0.22 µm filtered PBS, EV pellet from KG-1 or ME-1 was resuspended in 1.5 mL of 0.02 µm filtered PBS.

### Nanoparticle Tracking Analysis (NTA)

EV size and concentration were defined using NanoSight NS300 and data were processed using NTA 3.2 software (Malvern Panalytical Instrument, UK). D10 and D90 values, mode, mean and concentration were reported. D10 and D90 serve to identify the size range in which most of the particles are found and to weight the presence, proportion and importance of outsider particle sizes. Mode value indicates size of the highest number of particles measured in the middle of D10 and D90 range.

### Transmission Electron Microscopy (TEM)

An aliquot (20 μL) of EVs was applied to a Pioloform coated Nickel grid (200 mesh; TAAB Laboratories Equipment Ltd, Aldermaston, UK). Then, the grid was floated on the sample drop for 2 min and washed with 20 μL double distilled water drop. Negative staining was obtained with 200 μL of 2% w/v uranyl acetate solution (TAAB Laboratories Equipment Ltd). Samples were examined in a Philips Morgagni 282D TEM, operating at 60 kV. Electron micrographs of negatively stained samples were photographed on Kodak electron microscope film 4489 (Kodak Company, Rochester, NY, USA).

### Flow Cytometric Analysis of EV Samples

EV samples were analyzed on FACS CANTO II using DIVA software (Becton Dickinson, BD Biosciences, San Jose, CA, USA) as previously reported (18). Briefly, EV samples were labeled with 40 µM of 5-carboxyfluorescein diacetate-succinimidil ester (CFDA-SE, Sigma-Aldrich) and then were incubated with 100 ng of different conjugated antibodies for specific surface markers and their respective controls for 40 min at 37°C in a reaction volume of 30 µL. Specifically, phycoerythrin (PE) anti-CD14 (clone M5E2; BD), allophycocyanin (APC) anti-CD19 (clone SJ25C1; BD), PE anti-CD33 (clone P67.6; BD), APC anti-CD34 (clone 8G12; BD), APC anti-CD38 (clone HB-7; BD) and PE anti-CD117 (clone 104D2; BD) were used. After labeling, 3 µL of EV samples were transferred in TruCount tubes (BD) containing 400 µL of 0.02 µm filtered PBS. A total of 50,000 events were immediately acquired in low flow rate.

EV concentration (EVs/mL) was calculated with the formula EVs/mL=G_EV_/G_TC_*TC/V* DF (G_EV_= number of events CFSE^+^ in specific antibody positive EV gate, G_TC_=number of events in TruCount bead gate, TC= number of TruCount beads in single TruCount tube, V= sample volume used in the analysis, DF= sample dilution factor).

### EV Protein Quantification

Concentration of EV proteins was obtained by Bicinchoninic Acid (BCA) Protein Assay Kit (Thermo Scientific, Rockford, IL USA) as previously reported (19). The absorbance at 560 nm was measured using the VICTOR Nivo (Perkin Elmer, Waltham, MA, USA) and protein concentration was determined.

### CD34^+^ Isolation and Treatment With AML EVs

As source of CD34^+^ cells, UCB units were used. The percentage of CD34^+^ cells in UCBs is very low, around 0.1-0.5%, so many bags (~150 bags) were needed to purify CD34^+^ cells enough for our experiments. In addition, we point out that UCB bags were collected within 5 days before HSPC isolation. To obtain a concentration of all the white blood cells (buffy coats), the UCBs were centrifuged at 2800 rpm for 10 min. CD34^+^ cells were isolated from mononuclear cells by CD34 Microbead Kit using AutoMACS Pro separator (Miltenyi Biotec, Auburn, CA). The purity of isolated CD34^+^ cells routinely ranged between 90–95%.

All subjects provided written informed consent. This study complied with the Declaration of Helsinki and was approved by the Regional Committee for Medical and Health Research Ethics.

Immediately after isolation, CD34^+^ cells were seeded at 1x10^6^/mL in StemMACS HSC Expansion Media XF supplemented with StemMACS HSC Expansion Cocktail (Miltenyi Biotec) and treated with 200 µg of EVs or with the same volume of 0.02 µm filtered PBS (control) at 37°C and 5% CO_2_. It is noteworthy that this ratio could be considered comparable to the conditions in human AML BM since we found that the concentration of EVs in BM plasma of AML patients (with blasts in a range of 60-80%) was 364 ± 242 µg/ml. After 20 h of incubation with EVs or PBS, CD34^+^ were harvested and used for subsequent analysis.

### RNA Extraction From EVs and HSPCs

Total RNA was extracted from 300 µL of AML-EVs with Trizol reagent (Life Technologies, Carlsbad, CA USA) according to the manufacturer’s instructions.

Total RNA was extracted from CD34^+^ cells (4.5x10^4^ cells), treated or not with EVs, by RNA/DNA/PROTEIN Purification Plus Micro Kit (Norgen Biotek Corporation, Canada) according to the manufacturer’s instructions. Briefly, lysis buffer was added to cell pellets. Subsequently, sequential isolation of DNA and RNA was performed, and nucleic acids were eluted from spin columns in 50 μL elution buffer and nuclease-free water, respectively, and stored at -80°C until use. RNA from cells and EVs was quantified using the Nanodrop Spectrophotometer (Thermo Scientific, Wilmington, DE, USA).

### Microarray Experiments

HSPC-RNA quality was assessed by capillary electrophoresis on an Agilent 2100 Bioanalyzer (Agilent Technologies, Inc, Santa Clara, CA) using RNA 6000 Nano Chip Assay Kit (Agilent). Only samples with RNA integrity number (RIN) > 7 were used. For each sample, 300 ng of total RNA were used for gene expression experiments according to the Illumina TotalPrep RNA amplification kit protocol (Ambion Inc, Austin, TX), and cRNA was hybridized on Illumina HumanHT12 v4.0 Expression BeadChip array (Illumina Inc., San Diego, CA, USA), as previously described (20). BeadChip was scanned with an Illumina HiScanSQ system (Illumina Inc.).

### Reverse Transcription (RT) of RNA and Droplet Digital PCR (ddPCR)

RT of 10 ng EV-RNA was performed using TaqMan miRNA Reverse Transcription Kit and specific RT primers (Applied Biosystems, Foster City, CA, USA) for each miRNA, such as let-7a, let-7b, miR-150, miR-155, miR-181a, miR-125b and miR-21, following manufacturer’s instructions. Briefly, 2 μL of total EV-RNA were added into each tube containing RT reaction mixture and specific RT miRNA-primers in a total reaction volume of 20 μL. RT was performed for 30 min at 16°C, followed by 30 min at 42°C and 5 min at 85°C in the thermal cycler.

RT of 500 ng RNA from CD34^+^ cells, treated or not with EVs, was performed using High-Capacity cDNA Reverse Transcription Kit (Applied Biosystems) in 20 μL reaction volume following manufacturer’s instructions. RT was performed for 10 min at 25°C, followed by 120 min at 37°C and 5 min at 85°C in the thermal cycler.

The expression levels of EV-miRNAs and HSPC-mRNAs (CCL3, IL-1B, CSF2, GATA2, ZFP36 and CEBPA) were evaluated by QX200 ddPCR System (Bio-Rad Laboratories, Hercules, CA).

For EV-miRNAs, RT products were diluted 1:10 and 10 μL were added to a 2x ddPCR supermix for probe (Bio-Rad) plus 1 μL of 20x TaqMan probe for each miRNA (Applied Biosystems) in a final 20 μL reaction mix; for HSPC-mRNAs, cDNA was diluted 1:500 and 10 μL were added to a 2x ddPCR supermix for probe (Bio-Rad) plus 1 μL of 20x TaqMan probe for each mRNA (Applied Biosystems) in a 20 μL reaction mix.

Then, droplets were generated as previously described (19) and PCR amplification was carried out on a thermal cycler at 95°C for 10 min, then 40 cycles of 95°C for 15 seconds and 58°C (for miRNAs) or 60°C (for mRNAs) for 1 min, and finally 98°C for 10 min and 4°C infinite hold. A ramping rate of 2°C/sec was used in every step. Then, plate was read in QX200 droplet Reader (Bio-Rad) and analyzed using the Quantasoft TM version 1.7.4 software (Bio-Rad) obtaining the number of copies per microliter (n. copies/μL) for each sample.

### Flow Cytometric Analysis of CD34^+^ After EV Treatment

After treatment with AML EVs, CD34^+^ cells were washed and incubated with APC anti-CD10 (clone HI10a, BD), fluorescein isothiocyanate (FITC) anti-CD34 (clone 8G12; BD), PE-Cyanine 7 (PE-Cy7) anti-CD38 (clone HB-7; BD), APC-H7 anti-CD45RA (cloneHI100; BD Pharmingen), peridinin chlorophyll (PerCP) anti-CD90 (IgG1k, Clone 5E10; BD Pharmingen), PE anti-CD123 (clone7G3; BD Pharmingen), APC anti-C-X-C chemokine receptor type 4 (CXCR4, clone 12G5; BD Pharmingen) monoclonal antibodies, at room temperature for 15 min in the dark. After incubation, cells were washed and resuspended in PBS. Then, 10,000 events were acquired on FACS CANTO II and analyzed by DIVA software (BD).

### Colony-Forming Unit (CFU) Assay

One thousand and five hundred CD34^+^ cells treated or not with KG-1 and ME-1 derived EVs, were cultured in two 35 mm dishes in MethoCult Classic (Stem Cell Technologies, Vancouver, BC, Canada) according to the manufacturer’s instruction. All type of colony forming unit (CFU), specifically Burst-Forming-Unit Erythrocyte (BFU-E), CFU Granulocyte, Erythrocyte, Macrophage, Megakaryocyte (CFU-GEMM), CFU Granulocyte, Macrophage (CFU-GM) and CFU Macrophage (CFU-M), were visualized and counted with Axio microscope (Carl Zeiss Inc., Thornwood, New York) after being cultured in incubator at 37°C and 5% CO_2_ for 14 days.

### Migration Assay

Migration assay was performed using transwell plates 6.5 mm in diameter with 5 μm pore filters (Corning Costar, Cambridge, MA, USA). The upper chambers were loaded with 15×10^4^ CD34^+^ treated or not with EVs, in 150 μL of RPMI-1640 and the lower chambers with 600 μL of RPMI-1640 containing SDF-1 (Sigma Aldrich, St. Louis, Missouri, USA) at concentration of 200 ng/mL. After 4 h incubation at 37°C and 5% CO_2_, cells in the lower chambers were counted.

### Statistical Analysis

Results are shown as mean ± S.D. of at least three independent experiments. Mann–Whitney U-test was used to analyze two group comparisons in both miRNA and mRNA quantification, CXCR4 expression and migration assay. Cytofluorimetric analyses of HSPC populations and colony forming unit were carried out by two-way ANOVA followed by Sidak multiple comparisons. For all tests, a p-value <0.05 was taken as statistically significant (GraphPad Prism 6 software).

### Microarray Data Analysis

Gene expression analyses were performed by R well known environment (https://www.r-project.org/, https://bioconductor.org) (21). Raw data from Illumina HumanHT-12_V4_0_R2 microarray were normalized using neqc function in limma package (22) and low-quality annotation probes were excluded. Differentially expressed genes (DEGs) were obtained on the linear model fit of the microarray data and among them only those with adjusted p-value < 0.05 were kept. The enrichment analysis was carried out by the Gene Set Enrichment Analysis (GSEA) function in the clusterProfiler package (23) and on the base of the gene sets collections Hallmark, Pathway and Gene Ontology (GO) in Molecular Signatures Database (MSigDB) (24, 25). Some customized plots were processed by ggplot2 package (26). Microarray data are available at the Gene Expression Omnibus (GEO) repository under accession number GSE189492.

### MiRNA Pathways and Identification of mRNA Targets of MiRNAs

A list of selected AML-EV derived miRNAs was uploaded in the public available bioinformatic tool miRTargetLink Human (https://ccb-web.cs.uni-saarland.de/mirtargetlink/) (27) for enrichment in pathways and for interaction with gene targets. This tool provides validated targets (by strong or weak parameter options). The obtained gene target lists were intersected with down-regulated genes from gene expression analysis.

## Results

### Characterization of AML Derived-EVs

**Figure 1:**
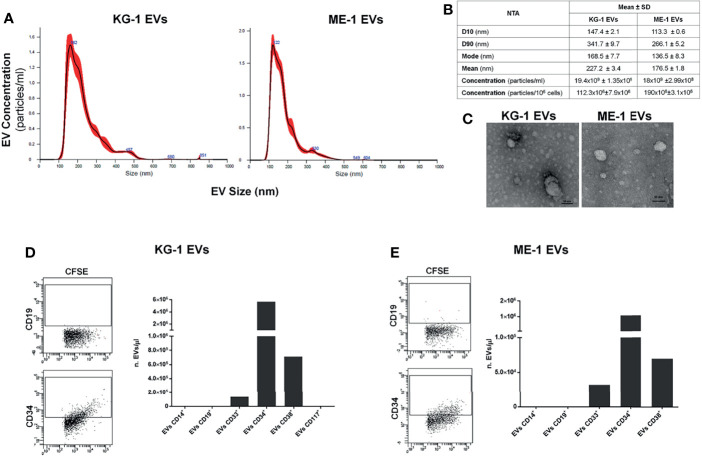
AML EV characterization. **(A)** Representative histograms of hydrodynamic size distribution profile of EVs from KG-1 and ME-1 cell lines measured by NTA. **(B)** NTA data are expressed as D10, D90, mode, mean and concentration (particles/mL and particles/1x10^6^ cells) as mean value ± standard error of AML derived-EVs. **(C)** Representative TEM images of AML cell line derived-EVs (image magnification: 100kX); horizontal bar indicates 50 nm. **(D, E)** Quantification as EV number for µL (nEVs/µL) of CD14^+^, CD19^+^, CD33^+^, CD34^+^, CD38^+^ and CD117^+^ EVs produced by KG1 and ME-1 cells. Representative cytometric dot plots of AML derived-CFSE^+^ EVs labeled for anti-CD19 and anti-CD34 surface markers were reported.

As source of AML-EVs we used EVs isolated from supernatant of two AML cell lines, KG-1 and ME-1. Their characterization by NTA analysis displayed a heterogeneous EV population, including both small and medium EVs, with a size range of 100-850 nm for KG-1 EVs and of 100-600 nm for ME-1 EVs (**Figure 1A**). In particular, 80% of KG-1 derived-EVs displayed a medium size ranging between 147.4 nm (D10) and 341.7 nm (D90); moreover, mode size of EVs was 168.5 nm. The concentration of KG-1 derived-EVs was 19.4x10^9^ ± 1.35x10^9^ for mL and 112.3x10^6^ ± 7.9x10^6^ for 10^6^ cells. As regarding ME-1 derived-EVs, most particles displayed a size range between 113.3 nm (D10) and 266.1 nm (D90), while mode size was 136.5 nm; EV concentration was 18x10^9^ ± 2.99x10^8^ for mL and 190x10^6^ ± 3.1x10^6^ for 10^6^ cells (**Figure 1B**).

AML EVs were also analyzed by TEM confirming a spheroid morphology and most of vesicles with a size lower than 50 nm (**Figure 1C**).

To further characterize AML EVs, we analyzed and quantified different surface antigens which are expressed on their origin cells. EV phenotyping showed that EVs, derived from both KG-1 and ME-1 AML cell lines, resulted positive for AML markers, like CD33, CD34 and CD38; instead, they resulted negative for CD14, CD19 and CD117 antigens (**Figures 1D, E** **)**. Moreover, this analysis showed a higher quantity of CD34 positive (+) and CD38^+^ AML EVs and lower amount of CD33^+^ EVs.

Finally, we analyzed EV miRNA content evaluating the presence of some miRNAs, including let-7a, let-7b, miR-125b, miR-150, miR-155, miR-181a, miR-128a and miR-21, which are involved in induction and regulation of AML. Interestingly, all miRNAs were detected and quantified, with different amounts, in EVs derived from both KG-1 and ME-1 cell lines (**Table 1**). Of note, these miRNAs are involved in the “Extracellular vesicle-mediated signaling in recipient cells” pathway, as reported by the bioinformatic tool miRTarget Link 2.0 (**Supplementary Table 1**).

**Table 1:** AML-EV miRNA quantification in KG-1 EVs and ME-1 EVs.

miRNAs	copies/µl	
	KG-1 EVs	ME-1 EVs
*hsa-let-7a*	79,4	22,3
*hsa-let-7b*	32,7	70,8
*hsa-miR-150*	15,8	9,9
*hsa-miR-155*	21,3	18,9
*hsa-miR-181a*	29,5	111,4
*hsa-miR-125b*	8,1	10,9
*hsa-miR-21*	3,1	1,8

### AML Derived-EVs Modified the Distribution of Myeloid Progenitors of HSPCs and Negatively Impaired Their Clonogenicity

**Figure 2:**
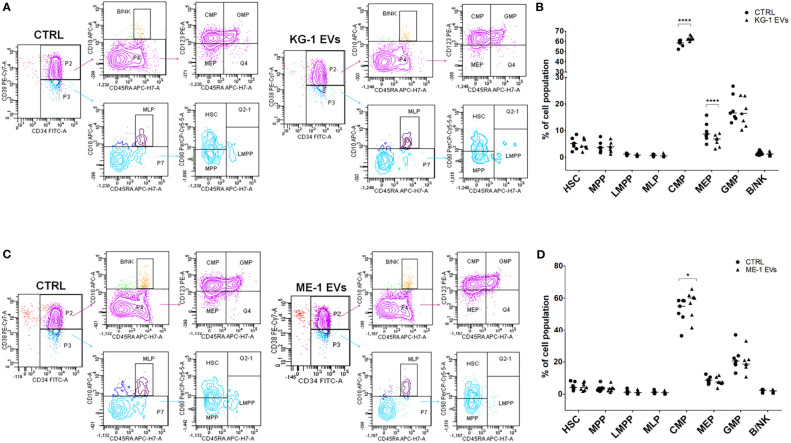
AML-EV effects on HSPC differentiation. **(A–C)** Representative cytofluorimetric dot plots of different stem progenitor populations [hematopoietic stem cells (HSC), multipotent progenitors (MPP), lymphoid-primed multipotent progenitors (LMPP), multi-lymphoid progenitors (MLP), common myeloid progenitors (CMP), granulocyte-macrophage progenitors (GMP), megakaryocyte-erythroid progenitors (MEP) and B and NK cell progenitors (B/NK)] treated or not (CTRL) with KG-1 and ME-1 derived-EVs for 20 h. **(B–D)** Percentage of cell populations after treatment with KG-1 and ME-1 derived-EVs. Each symbol represents a single experiment and horizontal bars represent median values. Statistically significant analyses are indicated by asterisks: *p < 0.05 and ****p < 0.0001.

We treated CD34^+^ cells with KG-1 and ME-1 derived-EVs or with PBS as control, for 20 h. To understand whether AML derived-EVs induced changes of HSPC differentiation, we analyzed by flow cytometer the frequency distribution of CD34^+^ progenitor cells treated or not with KG-1 or ME-1 EVs. We focused our attention on HSPC populations that include hematopoietic stem cells (HSC), multipotent progenitors (MPP), lymphoid-primed multipotent progenitors (LMPP), multi-lymphoid progenitors (MLP), common myeloid progenitors (CMP), granulocyte-macrophage progenitors (GMP), megakaryocyte-erythroid progenitors (MEP) and B and NK cell progenitors (B/NK). These populations were distinguished using a cytofluorimetric gating strategy as reported by Karamitros et al. (28) and described in **Table 2**. After 20 h of KG-1 EV treatment, cytofluorimetric analysis showed a significant increase of CMP and a decrease of MEP populations in treated cells respect to control (p<0.0001) (**Figures 2A, B** **)**. Likewise, the same trend for both populations was observed after treatment with ME-1 EVs (**Figures 2C, D** **)**. In addition, in ME-1 EV treatment, it was also observed a reduction of GMP population (**Figures 2C, D** **)**. Moreover, no difference was found for the other populations in both EV treatments except for a small decrease of HSC and LMPP in KG-1 EV treated CD34^+^ cells versus control. These data indicated that both KG-1 and ME-1 EVs induced an accumulation of less differentiate CMP with a consequent decrease of more differentiated progenitors MEP and GMP.

**Table 2:** HSPC populations and their immunophenotype.

HSPC populations	Cell immunophenotype
Hematopoietic Stem Cells (HSC)	CD34^+^ CD38^-^ CD90^+^ CD45RA^-^ CD10^-^
Multipotent Progenitors (MPP)	CD34^+^ CD38^-^ CD90^-^ CD45RA^-^ CD10^-^
Lymphoid-primed Multipotent Progenitors (LMPP)	CD34^+^ CD38^-^ CD90^-/lo^ CD45RA^+^ CD10^-^
Multipotent Lymphoid Progenitors (MLP)	CD34^+^ CD38^-^ CD90^-/lo^ CD45RA^+^ CD10^+^
Common Myeloid Progenitors (CMP)	CD34^+^ CD38^+^ CD123^+^ CD45RA^-^ CD10^-^
Megakaryocyte Erythroid Progenitors (MEP)	CD34^+^ CD38^+^ CD123^-^ CD45RA^-^ CD10^-^
Granulocyte Macrophage Progenitors (GMP)	CD34^+^ CD38^+^ CD123^+^ CD45RA^+^ CD10^-^
B/NK progenitors (B/NK)	CD34^+^ CD38^+^ CD90^-^ CD45RA^+^ CD10^+^

**Figure 3:**
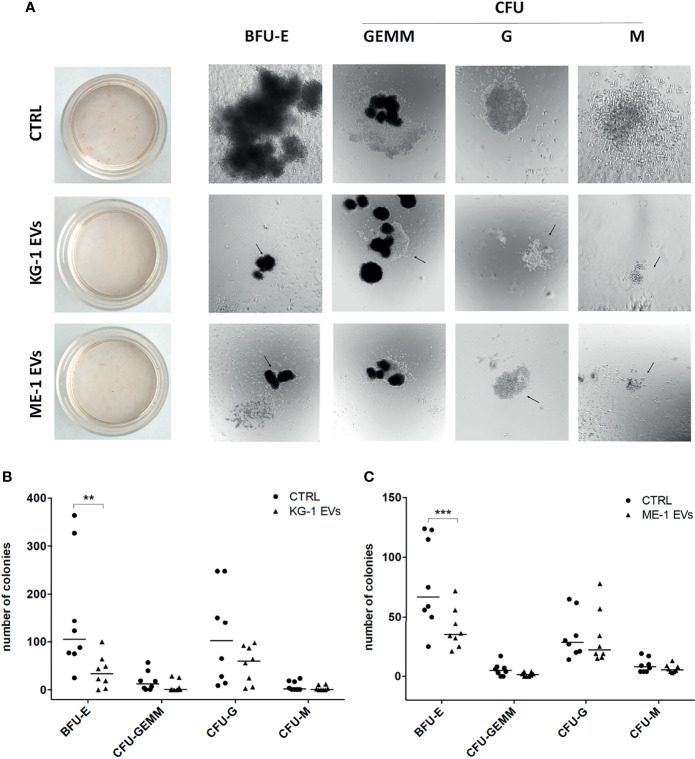
AML-EV effects on HSPC colony formation. **(A)** Representative images of Burst-Forming-Unit Erythrocyte (BFU-E), colony forming unit (CFU)- Granulocyte, Erythrocyte, Macrophage, Megakaryocyte (CFU-GEMM), CFU Granulocyte (CFU-G) and CFU Macrophage (CFU-M) after 14 days of culture of HSPCs treated or not (CTRL) with KG-1 orME-1 derived-EVs. **(B, C)** Number of different colonies counted after 14 days of culture of HSPCs treated or not with AML-EVs. Each symbol represents a single experiment and horizontal bars represent median values. Statistically significant analyses are indicated by asterisks: **p < 0.01 and ***p < 0.001.

To understand whether these changes had a repercussion on myeloid clonogenicity potential, we cultured CD34^+^ cells treated or not with AML-EVs in methylcellulose-based CFU assay. Of note, we observed that EV-treatment inhibited the formation of BFU-E, CFU-GEMM, CFU-G and CFU-M reducing their size and number (**Figure 3A–C**). Specifically, there was a significant decrease of BFU-E colony number in CD34^+^ cells treated with KG-1 EVs and with ME-1 EVs compared to their controls (p<0.01 and p<0.001 respectively) (**Figures 3B, C** **)**.

### HSPC Transcriptome Modification Induced by AML-EV Treatment

**Figure 4:**
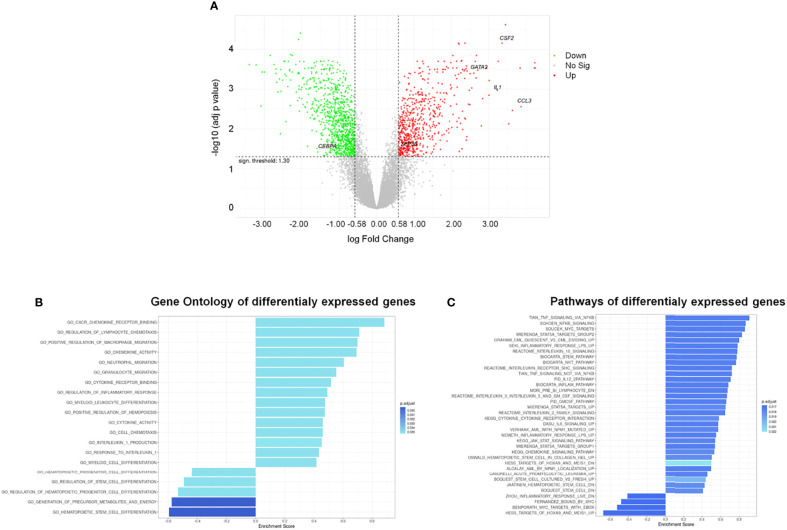
Differentially Expressed Genes (DEGs) and functional analysis of HSPCs treated with AML-EVs. **(A)** Volcano plot of all genes resulting from Gene Expression Profile (GEP) analysis in HSPCs treated with KG-1 EVs vs untreated cells; the x-axis represents the log fold-change value and y axis represents the statistical significance; the horizontal line indicates the significant threshold of DEG; the vertical lines represent the threshold of log fold change ≥ |0.58|; the labeled dots represent selected genes of interest. Significantly up- and down-regulated genes are highlighted in red and green, respectively. Gene ontology **(B)** and pathway **(C)** enrichment analysis performed on up- and down-regulated gene sets of HSPCs treated with AML-EVs. Left and right direction of bars represents down- and up-regulated gene sets, respectively.

To define possible changes due to AML-EVs on CD34^+^ gene expression, we analyzed transcriptome of KG-1 EV treated cells compared to untreated ones. Notably, we identified 1553 differentially expressed genes (DEGs) 630 up- and 923 down-regulated in treated vs untreated cells (adjusted p-value < 0.05 and absolute logFC value > 0.58) (**Supplementary Table 2** **;** **Figure 4A**).

The gene ontology (GO) enriched molecular pathway analysis and hallmark of DEGs revealed that they are involved in critical biological processes and molecular pathways, such as hematopoietic stem and progenitor cell differentiation, regulation of hematopoiesis (i.e., *CEBPA*, *GATA2* and *ZFP36)*, inflammatory response, cytokines activity (i.e., *CCL3, CSF-2/GM-CSF* and *IL1B*), chemotaxis, Stem pathways and IL-1B production (**Figures 4B, C** **;** **Supplementary Tables 3** **–** **5**).

To understand if down-regulated transcripts in HSPCs could be modulated by EV derived miRNAs, we questioned the miRTargetLink Human tool inserting the down-regulated mRNAs and EV-miRNAs. As expected, we found that every EV-miRNA targets multiple mRNAs while some mRNAs are common targets of more than one EV-miRNAs (i.e., *MYC* is target of let-7a/b and miR-155; *CBFB* is target of miR-155, and miR-125b (**Table 3** **;** **Supplementary Tables 6**).

**Table 3:** AML-EV miRNAs and their mRNA targets among the down-regulated genes in HSPCs treated with KG1-EVs.

EV-miRNAs	Copies/µL	Down-regulated mRNAs of HSPCs (Symbol)
*hsa-let-7a*	79.4	*ALDH7A1, AMD1, AURKB, C19orf53, CARHSP1, CCNB2, CDC7, CTPS1, DHX9, E2F2, EIF4A3, GATM, GLO1, LHFPL2, LRRC20, MARS2, MIEF1, MRPL12, MRPS2, MTHFD1, MYC, NME4, NOLC1, NUCB2, PDZD8, PGRMC1, PMPCA, POLR2D, PRIM2, PTGES2, QDPR, RPS24, RRM1, RRM2, SLC5A6, SNRPC, SRSF2, THEM6, UHRF1, UTP4, ZMYM3*
*hsa-let-7b*	32.7	*ADCK2, ALDH7A1, ALG3, AMD1, AURKA, AURKB, BIRC5, BZW2, C19orf53, CARHSP1, CBFB, CCNA2, CCNB2, CCND3, CDC25A, CDCA7, CDCA8, CENPV, CKB, CKS2, CLUH, COMMD9, COX7B, CPSF1, CTPS1, DCTPP1, DDX41, DHX33, DHX9, DNAAF5,DUSP23, E2F2, EEF1E1, EIF4A3, FAM136A, FANCD2, FEN1, GAPDH, GATM, GEMIN5, GGCT, GLO1, GPATCH4, GPHN, GPI, GSPT1, GTPBP3, HARS, HMGB1, HMGCS1, HSP90AA1, HSPA8, IMPDH1, IPO11, IPO4, ISOC2, KIFC1, LRRC20, MAP7, MARS2, MCM4, MCM7, MFSD3, MIEF1, MIPEP, MRPL12, MRPL37, MRPS11, MTFP1, MYC, NAA15, NCAPG2, NME4, NOLC1, NUCB2, NUP35, NUSAP1, PA2G4, PAAF1, PAFAH1B3, PDCL3, PDZD8, PFN1, PGRMC1, PIGU, PMPCA, POLD2, POLR2D, POLR2H, POLR3G, PPM1G, PRIM1, PRIM2, PRPS1, PSME3, PTGES2, PTTG1, QDPR, RPS24, RRM1, RRM2, RRP1B, RRP7A, SCD, SIGMAR1, SLC25A1, SLC25A19, SLC5A6, SNRPA, SUPT16H, TBRG4, THEM6, TIMM23, TTLL12, TUBA1B, TUBA1C, TUBB, TYMS, UCK2, UHRF1, WDR74*
*hsa-miR-150*	15.8	*AMD1, APEX2, ATP1B3, BIRC5, C21orf33, CACYBP, CARHSP1, CDK2, CENPM, CEP72, DLEU1, HMGB1, ISY1, MASTL, MRPL37, MS4A3, PDIA6, PDZD8, POLQ, PRIM1, PSMC1, RRP1B, TTLL12, WDR12*
*hsa-miR-155*	21.3	*ACOT7, AIFM1, AKR7A2, ALDH5A1, AURKA, AURKB, CARHSP1, CAT, CBFB, CCT2, CDK2, CDK4, CEP55, CHAF1B, COG2, COLGALT1, CSE1L, CTSA, CUTA, DHCR24, DIAPH3, DNAAF5, E2F2, EEF1E1, EIF2B5, EIF3G, EIF4E2, ERI1, EXOSC2, FDFT1, GAR1, GATM, GEMIN5, GMPS, H2AFY, HDHD5, HMGCS1, HSPB11, INTS10, KDELC2, KDM1A, KIF22, MARC1, MASTL, MCM8, MGST2, MRPL18, MSH6, MTAP, MYC, NCAPG, NOLC1, PEBP1, PGRMC2, POLE3, PPM1G, PSME3, PSMG1, PTEN, PUS7, RAB27B, RRM2, SCD, SLC25A19, SLC39A14, SRSF1, SRSF2, TRIP13, UQCRFS1*
*hsa-miR-181a*	29.5	*BRCA1, CEBPA, CTDSPL, EGR1, GPD1L, GTPBP3, H2AFY, HACD3, HNRNPAB, KIF2C, LPCAT1, METAP1, NCAPG, NPM3, PCLAF, PDIA6, PEBP1, PRDX3, PTEN, RAN, SCD, TUBB, ZFP36L2*
*hsa-miR-125b*	8.1	*ACLY, ACSS1, AURKB, CARHSP1, CBFB, CCNE1, CD320, CEBPA, DHX33, E2F2, HSPD1, IFRD2, LIPA, LSM4, LSS, MRTO4, NME2, NRARP, NUP205, NUP37, PPAT, PRDX2, PSMB1, RNASEH2A, RPL29, RPS7, RRP7A, SDHB, SHMT1, SLC19A1, SLC25A1, SMC2, TIMM23, UNG*
*hsa-miR-21*	3.1	*ACAT1, BRCA1, CCT6P1, CDC25A, E2F2, ECI2, FAM136A, FAM217B, FAM46A, FANCI, GPD1L, HMGB1, MSH6, MTAP, MYC, NCAPG, NFIB, OSBPL1A, PGRMC2, PTEN, PYM1, RPS7, SKP2, STUB1, TMEM147, TOP2A*

**Figure 5:**
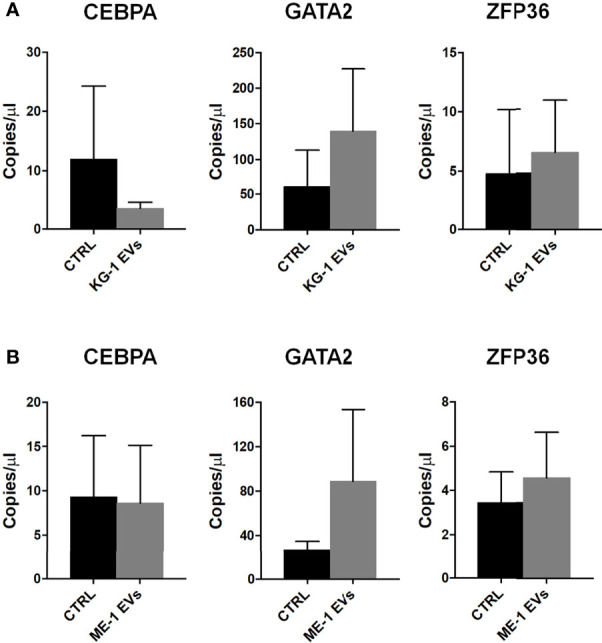
Hematopoiesis related mRNAs in HSPCs treated with AML-EVs. Absolute quantification (copies/μL) by ddPCR of *CEBPA*, *GATA2* and *ZFP36* mRNAs in HSPCs treated and not (CTRL) with KG-1 EVs **(A)** or ME-1 EVs **(B)**. Values represent results from three independent experiments (p > 0.05).

Subsequently, we analyzed by ddPCR several transcripts that are involved in hematopoiesis and in the differentiation of HSPCs such us *CEBPA*, *GATA2* and *ZFP36.* In particular, we found a decrease (< 3-fold changes) of *CEBPA* levels and an increase of *GATA2* (> 2 fold change) and *ZFP36* (1.3 fold changes) transcripts in HSPCs treated with KG-1-derived EVs (**Figure 5A**). Similar results were obtained treating HSPCs with ME-1 derived-EVs (**Figure 5B**).

### AML Derived-EVs Induced the Production of Inflammatory Cytokines, Reduced CXCR4 Expression Levels and Impaired SDF-1 Mediated Migration of HSPCs

**Figure 6:**
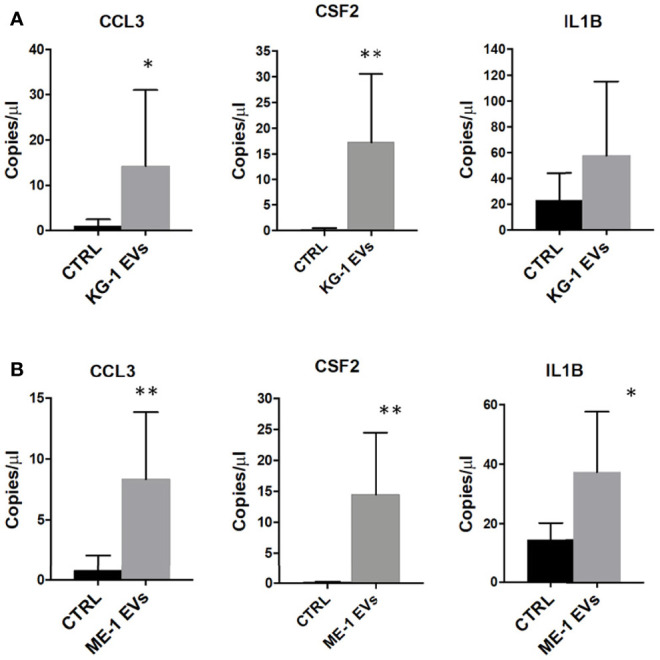
Inflammatory cytokines mRNAs in HSPCs treated with AML-EVs. Absolute quantification (copies/μL) by ddPCR of *CCL3*, *CSF2* and *IL1B* in HSPCs treated or not (CTRL) with KG-1 **(A)** or ME-1 **(B)** derived-EVs. The bar-graphs represent mean + SD from three independent experiments. Statistically significant analyses are indicated by asterisks: *p < 0.05 and **p < 0.01.

Gene Ontology (GO) analysis of up-regulated genes in HSPCs treated with KG-1 EVs classified inflammatory response and cytokine activity as the most activated functions (**Figure 4B** and **Supplementary Table 3**). We paid attention on CCL3, CSF-2 and IL1B cytokines to explore their expression in HSPCs after AML-EV treatment. A high expression level of *CCL3, CSF2* and *IL1B* mRNA was found in HSPCs treated with KG-1 EVs (p<0.05 for *CCL3*, p<0.01 for *CSF2*). In particular, the increased expression of *CCL3* and *CSF2* was >4 and >90 folds, respectively, while *IL1B* levels was >2 fold in treated HSPCs respect to untreated ones (**Figure 6A**). Similar results were obtained treating HSPCs with ME-1 derived-EVs (**Figure 6B**).

**Figure 7:**
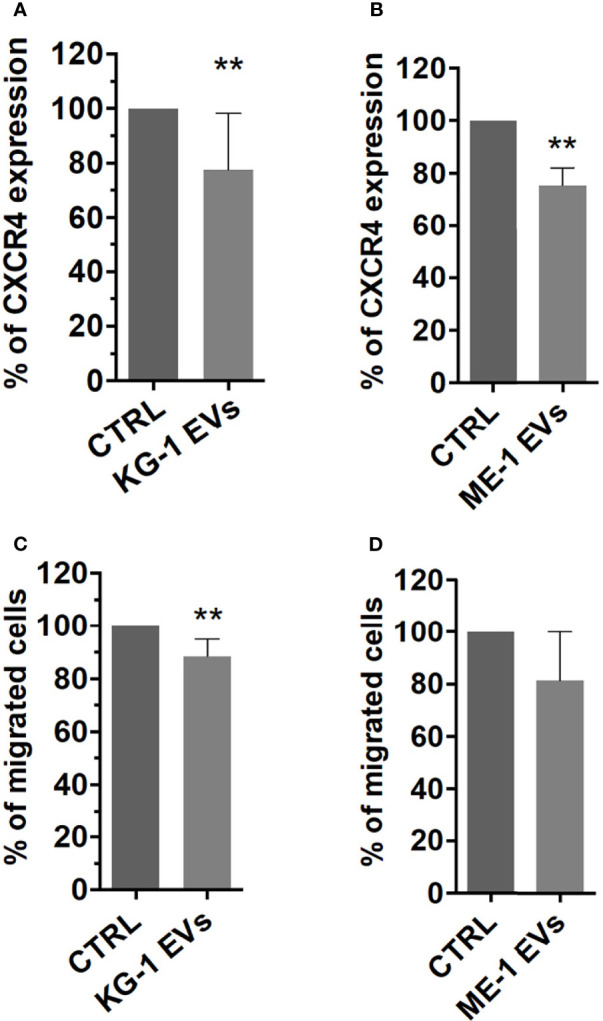
CXCR4 expression and SDF-1 mediated migration in HSPCs treated with AML-EVs. Percentage of CXCR4 expression on HSPCs treated or not with KG-1 **(A)** or ME-1 EVs **(B)**. Percentage of migrated cells after treatment of HSPCs with KG-1 **(C)** or ME-1 EVs **(D)**. The bar-graphs represent mean + SD from three independent experiments. Statistically significant analyses are indicated by asterisks: **p < 0.01.

BM homing and migration of HSPCs are regulated by the response to SDF-1, which in turn depends on the levels of CXCR4 expressed on HSPC surface (29). Therefore, to assess the effect of AML derived-EVs on CD34^+^ SDF-1 mediated migration, firstly, we evaluated CXCR4 receptor expression on CD34^+^ cells treated with EVs observing a significant decrease of its levels on EV treated cells respect to control (**Figures 7A, B** **)**. Subsequently, CD34^+^ cells treated or not were seeded in cell culture trans-well containing SDF-1 or medium. We observed a decrease of CD34^+^ migrated cells following treatment with EVs from both AML cell lines (**Figures 7C, D**); in particular, KG-1 EVs induced a significant reduction of CD34^+^ cell migration (p<0.01) and the same trend was observed in ME-1 EV CD34^+^ treated cells (**Figures 7C, D** **)**.

## Discussion

In the leukemic context, there is a considerable interest to study hematopoietic dysregulation during the conversion from normal BM microenvironment to leukemic niche. Different reports, using a combination of *in vitro* and *in vivo* studies, have demonstrated that AML blasts and their EVs are able to alter both BM composition and function (16). With this assumption, in this work we used EVs isolated from two AML cell lines, KG-1, an erythroleukemia, and ME-1, an acute myelomonoblastic leukemia with eosinophilia, to treat HSPCs with the aim to study possible changes induced by EV treatment.

AML derived-EV characterization identified the presence of a heterogeneous population of small and medium EVs with a size range between 50-850 nm for KG-1 EVs and 50-600 nm for ME-1 EVs. One million of AML cells, KG-1 or ME-1, released 112x10^6^ and 190x10^6^ EVs, respectively, after 48 h of culture. Furthermore, according to previous studies, we showed that AML-EVs display myeloid leukemia associated antigens, like CD34, CD33 and CD38 (30, 31), and carry several miRNAs (11, 32). Regard the EV content, we and others reported that AML EVs carried miRNAs, such as let-7a, let-7b, miR-125b, miR-150, miR-155, miR-181a and miR-21, which are involved in the pathogenesis of hematological malignancies (33, 34), including AML. In general, it is meanwhile well known that the EV content, including protein, RNA and surface marker composition, is probably strongly dependent on cell source, cells’ activation status and multiple other parameters. Since EVs usually show similar surface profiles than origin cells, analysis of EV surface signatures in biological fluid has the potential to reveal changes occurring, in terms of abundance, frequency and behavior, in the respective origin cells. Thus, it is a promising approach to identify EV surface markers and/or miRNA profiles that correspond to certain diseases such as leukemia; in fact, recently, EVs could also be used as biomarkers for diagnosis and therapy decision making (18). In this context, we are able to define, in the culture supernatant and in the serum, the concentration of specific AML-EVs including CD34^+^EVs, CD38^+^EVs and CD33^+^EVs.

This work highlighted that AML-derived EVs directly modified HSPCs, functionally compromising them and inducing a transcriptome upheaval. In fact, gene expression profile of CD34^+^ cells treated with KG-1 EVs showed 630 up- and 923 down-regulated genes in treated HSPCs respect to control. This effect was also confirmed through the evaluation of some up- and down-regulated genes on CD34^+^ treated with EVs isolated from the other cell line ME-1, phenotypically and molecularly different from KG-1. We analyzed gene expression differences by canonical enrichment gene sets evaluating: (i) gene ontology, (ii) pathways and (iii) hallmark. All these analyses highlighted that the EV induced-transcriptome upheaval mediates the inhibition of normal hematopoiesis (GATA2, ZFP36 and relative pathways).

Interestingly, we found that some down-regulated genes were targets of miRNAs contained in AML-EVs; this allowed us to hypothesize that such miRNAs, with other components of EVs, likely directly participate to modify the transcriptome of recipient cells. Further functional studies are warranted to identify the role(s) of specific EV components in this process. Of note, the identified miRNAs in AML-EVs are also involved in the “extracellular vesicle-mediated signaling in recipient cells” pathway. Our hypothesis supported by literature data reporting that AML-EVs contain RNA, DNA and proteins involved in the pathobiology of AML and that their signaling induces change in the proliferative, angiogenic and migratory characteristics of stromal cells and HSPCs (13). Recently, we demonstrated that the transfer of specific miRNAs by multiple myeloma (MM)-EVs in HSPCs also occur in MM setting reporting their increased levels in HSPCs after 20 h of MM-EV treatment (19).

Combining HSPC down-regulated mRNAs with EV derived-miRNAs, we noted that *CEBPA, CBFB* and *MYC* transcripts are involved in hematopoietic development/differentiation, as also reported in GO. In particular, CEBPA is a transcription factor mainly involved in myeloid development and an indispensable factor for the initiation of AML. Its deletion in BM of adult mice failed to generate granulocyte/monocyte progenitors and resulted in a complete block of neutrophilic development (35). We found *CEBPA* reduced and, more interestingly, it was reported to be target of miR-125b (36). Core-binding factor subunit beta (*CBFB*) is target for miR-125b whose over-expression in normal HSPCs promotes their transformation into malignant cells (33). In addition, *CBFB* was also identified to be a target of miR-155, carried by our AML-EVs. MYC nuclear protein is required for the correct balance between self-renewal and differentiation of HSPCs (37) as a downstream mediator of Hoxb4 (38). Of note, we found that Hoxb4 was also down-regulated by AML-EVs.

Among the up-regulated genes also involved in hematopoietic development, we focused on *GATA2* and *ZFP36*. Transcription factor GATA2 is essential for the development of erythroid and megakaryocytic lineages. Of note, in AML setting, GATA2 induces the production of chemokine CXCL2 and cytokine IL-1B and high GATA/CXCL2 expression predicts poor prognosis in AML (39). Interestingly, we found that EV induced up-regulation of GATA2 was accompanied by an increase in the amount of IL-1B.

ZFP36 is a member of RNA-binding proteins (RBPs) increasingly appreciated as being essential for normal hematopoiesis, and it is understood to play fundamental roles in hematological malignancies by acting as oncogene or tumor suppressor (40). Recently, it was investigated RBP cooperation in the control of the inflammatory response (41). Of note, in our setting, HSPCs showed reduced levels of Z*FP36* and high pro-inflammatory GM-CSF (also known as CSF2) levels.

The EV induced-transcriptomic “upheaval” was accompanied by an HSPC functional impairment. Indeed, analyzing the frequency distribution of CD34^+^ progenitor cells (HSC, MPP, LMPP, MLP, CMP, GMP, MEP, B/NK) treated or not with AML EVs, we observed a differentiation deregulation. We found an increase of less differentiated CMP and a decrease of more differentiated myeloid progenitors MEP and GMP. Moreover, no difference was found for the other populations in both AML-EV treatment except for a small decrease of HSC and LMPP in CD34^+^ cells treated with KG-1 EVs versus control. This indicated that AML-EVs particularly induced an accumulation of less differentiated myeloid progenitors at the expense of more differentiated progenitors and had no or little effect on the other populations. Moreover, to confirm the suppression of myeloid differentiation, our study showed a strong reduction of different myeloid colony-forming unit, BFU-E, CFU-GEMM, CFU-GM and CFU-M, in term of size and number. Our data were in agreement with Zhao et al., which demonstrated that treatment of HSCs with AML exosomes containing miR-4532 reduced CFU formation and increased the expression of DKK1, a hematopoietic inhibiting factor (3). We hypothesized that AML-EVs could contribute to the “leukemic transformation” of healthy HSPCs by fueling disease progression causing an initial differentiation block and making HSPCs more sensitive to subsequent “modifications” that will transform them into leukemic blasts. In fact, the accumulation of immature cells that lack normal differentiation is one of the peculiarities of AML, in which the deregulation of hematopoiesis results in BM insufficiency (42). Further studies will need to confirm this hypothesis.

GO analysis also identified functions like inflammation, cytokine activity and cellular migration/chemotaxis supposing a stimulation of HSPC mobilization and/or attraction from other cell types. We found that AML-EVs induced an over-expression of *CCL3*, *IL-1B* and *CSF2/GM-CSF* cytokines in HSPCs, at the transcriptional level. Although we did not directly assess cytokines levels, we are confident that the increased transcriptional expression correspond to an increased production of these cytokines.

CCL3, also known as Macrophage Inflammatory Protein-1α (MIP-1α), can induce chemotactic mobilization of monocyte-lineage cells and lymphocytes into inflammatory tissues. In leukemia, *CCL3* can induce multiple processes that support the dominant proliferation of tumor cells: (*i*) conversion of normal niche cells to leukemia-adapted cells; (*ii)* selective inhibition of normal HSPCs; (*iii)* mobilization of normal HSPCs from BM (43). CCL3 expression is elevated in the majority (~75%) of primary AML samples (44). In our setting, AML-EVs induced a 4-fold-increase of *CCL3* expression in HSPCs respect to control.

IL-1B is a pleiotropic inflammatory cytokine whose over-expression is a common event in patients with hematological malignancies (45). It is mainly produced by myeloid cells (46) and modulates HSC function. In preclinical models, it promotes a partial HSC differentiation into the myeloid lineage (47). Our treatment with AML-EVs induced an increased amount of IL-1B in HSPCs as reported by Huan et al. (7). Interestingly, IL-1B promotes the expansion of AML progenitor cells suppressing the colony formation ability of healthy progenitors and enhances the production of other pro-inflammatory cytokines, such as CCL3 and IL-6 from AML progenitors (48).

CSF2, commonly known as GM-CSF, is an autocrine/paracrine cytokine that stimulates the multipotent progenitor cells. It is known as a hematopoietic growth factor involved in the survival, proliferation and differentiation of various myeloid cells from hematopoietic progenitor cells (49). Recently, it has been reported that it is actively secreted by stem cells, in response to various types of injury (50, 51). Our results indicated an increase of 90 folds of CSF2/GM-CSF in EV treated-HSPCs.

We hypothesized that, in the leukemic setting, there is an interdependent co-regulation of pro-inflammatory cytokines, such as IL-1, CCL3 and GM-CSF, with this last as part of a positive feedback “loop” involving communication intra leukemic HSPCs by increasing the number of multipotent progenitor cells which, in turn, increase the production of GM-CSF that, with an autocrine loop, supports them and among leukemic HSPCs, monocytes/macrophages and neighboring BM cell populations, such as fibroblasts, endothelial cells etc.

Overall, we believe that AML cells, through EVs, induce HSPCs to release cytokines and grow factors which contribute to generate an inflammatory environment that drives leukemia (52, 53) and supports “leukemic-like” HSPC growth.

Healthy HSPCs are localized into BM microenvironment known as the stem cell “niche”, where SDF-1 and its receptor CXCR4 contribute to BM stem cell homing, migration, proliferation and quiescence (54). In this study, AML-EVs induced a reduction of surface expression of CXCR4, driving HSPCs to be less attracted and therefore to migrate less towards SDF-1. Our data are in accordance with Huan et al. showing that AML exosomes directly reduce CXCR4 and c-Kit expression, the colony forming capacity, and repress several hematopoietic transcription factors of HSPCs (7, 55).

Of note, we think that AML-EV treatment induced specific effects on HSPCs. In fact, performing similar experiments in another neoplastic context, such as MM, we obtained different results in HSPC differentiation and related functions (19).

## Conclusions

**Figure 8:**
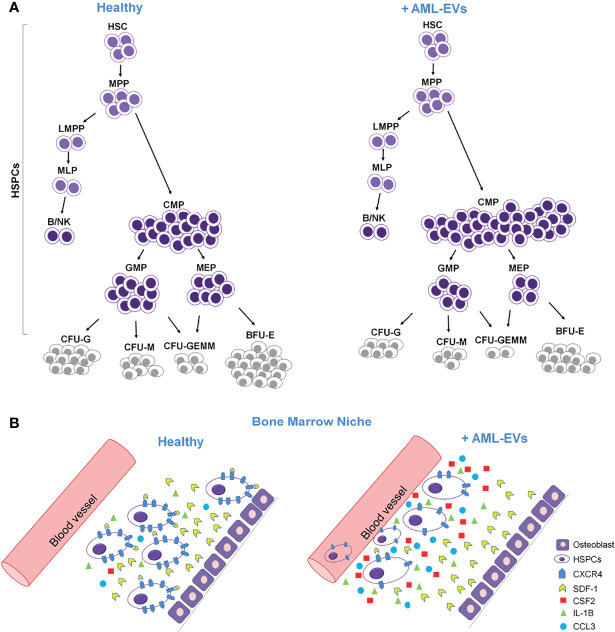
Cartoon describing the effects of AML-EVs on HSPCs. **(A)** AML-EVs inhibit the differentiation of HSPCs inducing: (1) an increase of common myeloid progenitors (CMP), (2) a decrease of granulocyte macrophage progenitors (GMP) and megakaryocyte erythroid progenitors (MEP), and (3) a decrease of myeloid colonies [Burst-Forming-Unit Erythrocyte (BFU-E), colony forming unit (CFU)- Granulocyte, Erythrocyte, Macrophage, Megakaryocyte (CFU-GEMM), CFU Granulocyte (CFU-G) and CFU Macrophage (CFU-M)]. **(B)** Schematic model of both healthy and leukemic BM niche. AML-EVs induce an increase of inflammatory cytokines such as CCL3, CSF2 and IL-1B, and a decreased expression of CXCR4 accompanied by a reduced SDF-1 mediated attraction of HSPCs.

In conclusion, our study suggests that leukemia derived EVs functionally compromise healthy HSPCs causing (*i)* an accumulation of less differentiated myeloid progenitors; (*ii)* a reduction of myeloid colony formation; (*iii)* an increased mRNA levels of inflammatory cytokines and growth factors and, finally; (*iv)* a reduction of CXCR4 expression and SDF1-mediated migration (**Figures 8A, B** **)**. This study proves that AML-EVs directly suppress normal hematopoiesis to favor leukemic environment and tumor growth. Moreover, it could provide novel rational for a therapy in AML.

## Data Availability Statement

The datasets presented in this study can be found in online repositories. The names of the repository/repositories and accession number(s) can be found below: https://www.ncbi.nlm.nih.gov/geo/, GSE189492.

## Ethics Statement

This study was approved by Ethic Committee of both Istituto di Ricovero e Cura a Carattere Scientifico – Centro di Riferimento Oncologico della Basilicata (IRCCS- CROB), and Casa Sollievo della Sofferenza Hospital. The patients/participants provided their written informed consent to participate in this study.

## Author Contributions

ST and IL performed *in vitro* EV/HSPC experiments, flow cytometer analysis, colony assay, gene expression profile, and digital PCR. DL performed the EV extraction and HSPC isolation. GC performed statistical and GEP analyses. ADS performed TEM analysis of EVs. MS selected and supplied UCB bags. AS critically revised the manuscript. AC and LDL designed, coordinated the research plan and wrote the final version of the manuscript. All authors contributed to the article and approved the submitted version.

## Funding

This work was supported by the Italian Ministry of Health (IT-MOH) through funding of Ricerca Corrente 2020.

## Conflict of Interest

The authors declare that the research was conducted in the absence of any commercial or financial relationships that could be construed as a potential conflict of interest.

## Publisher’s Note

All claims expressed in this article are solely those of the authors and do not necessarily represent those of their affiliated organizations, or those of the publisher, the editors and the reviewers. Any product that may be evaluated in this article, or claim that may be made by its manufacturer, is not guaranteed or endorsed by the publisher.
